# Adherence of Nontypeable Haemophilus influenzae to Cells and Substrates of the Airway Is Differentially Regulated by Individual ModA Phasevarions

**DOI:** 10.1128/spectrum.04093-22

**Published:** 2022-12-13

**Authors:** Preeti Garai, John M. Atack, Brandon M. Wills, Michael P. Jennings, Lauren O. Bakaletz, Kenneth L. Brockman

**Affiliations:** a Department of Microbiology and Immunology, Medical College of Wisconsin, Milwaukee, Wisconsin, USA; b Institute for Glycomics, Griffith University, Gold Coast, Queensland, Australia; c School of Environment and Science, Griffith University, Gold Coast, Queensland, Australia; d Abigail Wexner Research Institute, Center for Microbial Pathogenesis, Nationwide Children’s Hospital, Columbus, Ohio, USA; e College of Medicine, Department of Pediatrics, The Ohio State University, Columbus, Ohio, USA; University of North Dakota

**Keywords:** ModA phasevarion, NTHi, adherence, respiratory tract substrates

## Abstract

Adherence of nontypeable Haemophilus influenzae (NTHi) to the host airway is an essential initial step for asymptomatic colonization of the nasopharynx, as well as development of disease. NTHi relies on strict regulation of multiple adhesins for adherence to host substrates encountered in the airway. NTHi encode a phase-variable cytoplasmic DNA methyltransferase, ModA, that regulates expression of multiple genes; a phasevarion (phase-variable regulon). Multiple *modA* alleles are present in NTHi, in which different alleles methylate a different DNA target, and each controls a different set of genes. However, the role of ModA phasevarions in regulating adherence of NTHi to the host airway is not well understood. This study therefore sought to investigate the role of four of the most prevalent ModA phasevarions in the regulation of adherence of NTHi to multiple substrates of the airway. Four clinical isolates of NTHi with unique *modA* alleles were tested in this study. The adherence of NTHi to mucus, middle ear epithelial cells, and vitronectin was regulated in a substrate-specific manner that was dependent on the ModA allele encoded. The adhesins Protein E and P4 were found to contribute to the ModA-regulated adherence of NTHi to distinct substrates. A better understanding of substrate-specific regulation of NTHi adherence by ModA phasevarions will allow identification of NTHi populations present at the site of disease within the airway and facilitate more directed development of vaccines and therapeutics.

**IMPORTANCE** Nontypeable Haemophilus influenzae (NTHi) is a predominant pathogen of the human airway that causes respiratory infections such as otitis media (OM) and exacerbations in the lungs of patients suffering from chronic obstructive pulmonary disease (COPD). Due to the lack of a licensed vaccine against NTHi and the emergence of antibiotic-resistant strains, it is extremely challenging to target NTHi for treatment. NTHi adhesins are considered potential candidates for vaccines or other therapeutic approaches. The ModA phasevarions of NTHi play a role in the rapid adaptation of the pathogen to different environmental stress conditions. This study addressed the role of ModA phasevarions in the regulation of adherence of NTHi to specific host substrates found within the respiratory tract. The findings of this study improve our understanding of regulation of adherence of NTHi to the airway, which may further be used to enhance the potential of adhesins as vaccine antigens and therapeutic targets against NTHi.

## INTRODUCTION

Nontypeable Haemophilus influenzae (NTHi) is a host-adapted mucosal pathogen that colonizes the human nasopharynx asymptomatically from early childhood (1–3). However, as a predominant pathogen of the human respiratory tract, NTHi also causes infections at multiple sites within the airway. These include otitis media (OM), an inflammatory disease of the middle ear (4), and exacerbations in the lungs of patients with chronic obstructive pulmonary disease (COPD) and cystic fibrosis (CF) (5, 6). NTHi can also cause bronchitis, sinusitis, and community-acquired pneumonia (7–9). Approximately 10% of the human population (over 700 million people) is affected by OM, which can result in hearing loss and negatively impact learning. The complications associated with OM lead to the death of more than 20,000 people per year globally, with the highest morbidity and mortality in children under the age of 5 (10). COPD is the third leading cause of deaths worldwide (11, 12). There are currently no licensed vaccines available against NTHi.

Adherence of NTHi to the airway epithelium is the primary step in the colonization of the human nasopharynx and is important in the development of disease (13–18). For instance, ascension of NTHi from the nasopharynx to the middle ear, a required step in the development of OM, depends on the adherence of NTHi to mucus present within the Eustachian tube (19). NTHi expresses multiple surface-associated adhesins that specifically bind to cognate host receptor proteins, extracellular matrix components (ECMs), or mucus to facilitate adherence at different sites of the host airway (19–23). Because NTHi lacks a capsule, adhesins are more accessible on the surface of these bacterial cells than in their encapsulated counterparts. This accessibility also makes these surface-exposed adhesins ideal vaccine candidates for protection against NTHi-induced infections (24–26).

NTHi, like several other host-adapted mucosal pathogens, encodes a phase-variable DNA methyltransferase, ModA (27–31). Phase variation is a rapid and reversible switching of expression of a protein. In the case of *modA*, phase variation is due to simple DNA sequence repeats (SSRs) within the gene’s open reading frame. Variation in length of this SSR tract results in the absence (status “OFF”) or the expression (status “ON”) of the encoded ModA protein. This biphasic OFF-ON switching of methyltransferase expression results in genome wide methylation differences and the regulation of multiple genes via epigenetic mechanisms forming a phase-variable regulon, or phasevarion (31). Each NTHi strain encodes a single *modA* allele, with 22 *modA* alleles currently characterized in NTHi and the pathogenic Neisseria (28, 32, 33). The specificity of the ModA protein is dictated by the highly variable target recognition domain (TRD). Changes in the TRD alter the specificity of the methyltransferase and result in methylation of different DNA target sequences. Therefore, different *modA* alleles regulate different phasevarions. Of the 22 *modA* allelic variants reported for NTHi so far, *modA2* is the most prevalent among multiple clinical isolate collections (27, 32, 33). Analysis of phase-variable *modA* allele distribution across multiple NTHi strain collections revealed that two-thirds of the OM isolates encode just one of five *modA* alleles: *modA2*, *modA4*, *modA5*, *modA9*, and *modA10* (33); the majority of the COPD isolates contained a *modA2*, *modA4*, or *modA5* allele (32). Each ModA phasevarion regulates a unique set of genes, including many adhesins and/or potential vaccine antigens (32, 33). For example, the ModA2 phasevarion has been shown to regulate multiple disease-related phenotypes, including resistance to oxidative stress (34), biofilm formation (35), and virulence in the chinchilla model of experimental OM (36, 37). The ModA4 and ModA5 phasevarions are known to play distinct roles in the pathogenesis of NTHi, such as evasion from opsonization and susceptibility to antibiotics (33). However, the specific role of these different ModA phasevarions in adherence of NTHi to the host airway has not been investigated. We therefore sought to address the roles of the ModA2, ModA4, ModA5, and ModA9 phasevarions in the adherence of NTHi to various cellular and noncellular substrates encountered by NTHi within the airway.

## RESULTS

### ModA2 phasevarion regulates adherence of NTHi to mucus.

The NTHi clinical strains 723, C486, 477, and 1209 contain the unique *modA* alleles *modA2*, *modA4*, *modA5*, and *modA9*, respectively (33), and were therefore selected for this study to represent each of these phasevarions. These strains were genetically modified to permanently lock the expression of ModA in the OFF or ON status and thus generated *modA* locked OFF and *modA* locked ON variants of each strain (Table 1). The use of these locked variants, here referred to as *modA* OFF and *modA* ON variants, allowed the assessment of the direct effect of each *modA* status on the adherence of NTHi without the confounding effects of *modA* phase variation during the phenotypic assays.

**TABLE 1:** Bacterial strains^*a*^

Strain	Reference
NTHi 723 *modA2* locked OFF	34
NTHi 723 *modA2* locked ON	34
NTHi C486 *modA4* locked OFF	This study
NTHi C486 *modA4* locked ON	This study
NTHi 477 *modA5* locked OFF	This study
NTHi 477 *modA5* locked ON	This study
NTHi 1209 *modA9* locked OFF	This study
NTHi 1209 *modA9* locked ON	This study
NTHi 723 Δ*ompE modA2* locked OFF	This study
NTHi 723 Δ*ompE modA2* locked ON	This study
NTHi 723 Δ*hel modA2* locked OFF	This study
NTHi 723 Δ*hel modA2* locked ON	This study
NTHi 723 Δ*hap modA2* locked OFF	This study
NTHi 723 Δ*hap modA2* locked ON	This study

a NTHi, nontypeable Haemophilus influenzae.

**FIG 1:**
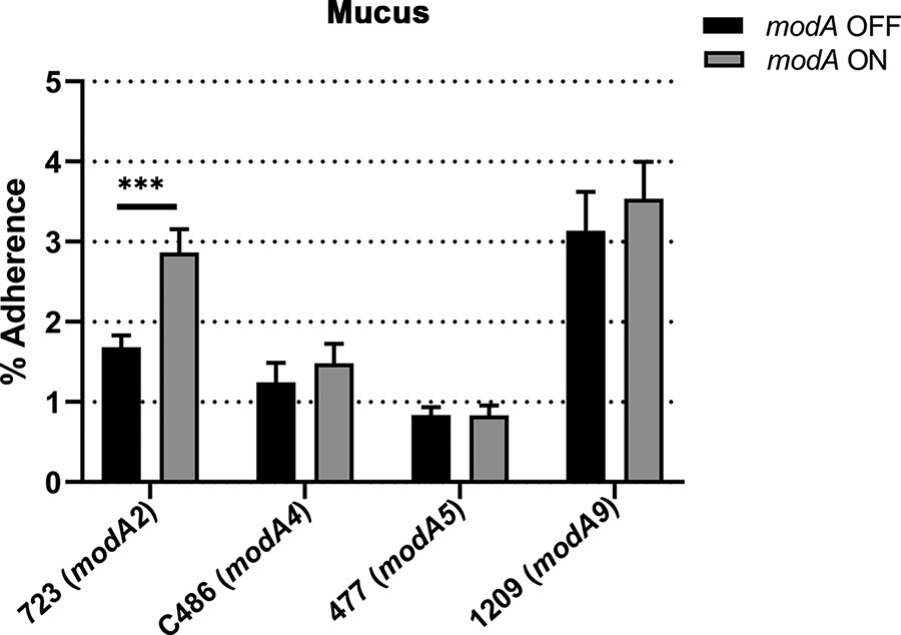
Adherence of NTHi *modA* locked variants to mucus. The data are shown as percentages of adherent bacteria relative to inoculum after 1 h. *modA2* ON adhered significantly better than *modA2* OFF. There was no significant difference between the variants of *modA4*, *modA5*, or *modA9*. *modA2*, *n* = 18; *modA4*, *n* = 15; *modA5*, *n* = 11; *modA9*, *n* = 14. ***, *P < *0.001 (Student’s *t* test).

Mucus coats the surface of the airway epithelium, and as such serves as an initial substrate for NTHi adherence and can further facilitate movement of the pathogen to different sites within the airway (19, 20, 22, 23, 38). Therefore, adherence to mucus is crucial for NTHi colonization and pathogenesis. The respiratory tract epithelium is composed of a variety of cells, which include goblet cells that produce mucus and ciliated cells that propel the mucus through the airway (39). Normal human primary bronchial-tracheal epithelial cells (nhPBTEs) were grown at an air-liquid interface (ALI) to induce formation of differentiated polarized cells that mimic the pseudostratified epithelium of the respiratory tract (40). Mucus produced by these polarized nhPBTEs was collected and used to assess the adherence of each *modA* variant. Bacteria were incubated in mucus-coated wells for 1 h, and adherent bacteria were enumerated. The *modA2* OFF variant of strain 723 adhered to mucus significantly less than the *modA2* ON variant (Fig. 1; *P* < 0.001). However, there was no significant difference between the *modA* ON-OFF variants of strains C486 (*modA4*), 477 (*modA5*), or 1209 (*modA9*). Interestingly, strain 477 adhered the least, and strain 1209 was the most adherent to mucus irrespective of *modA* status (Fig. 1). Thus, it appears that ModA2 regulates factors important in adherence of NTHi to mucus in addition to other ModA-independent strain differences.

### Adherence of NTHi to human bronchial-tracheal epithelial cells is not regulated by ModA.

**FIG 2:**
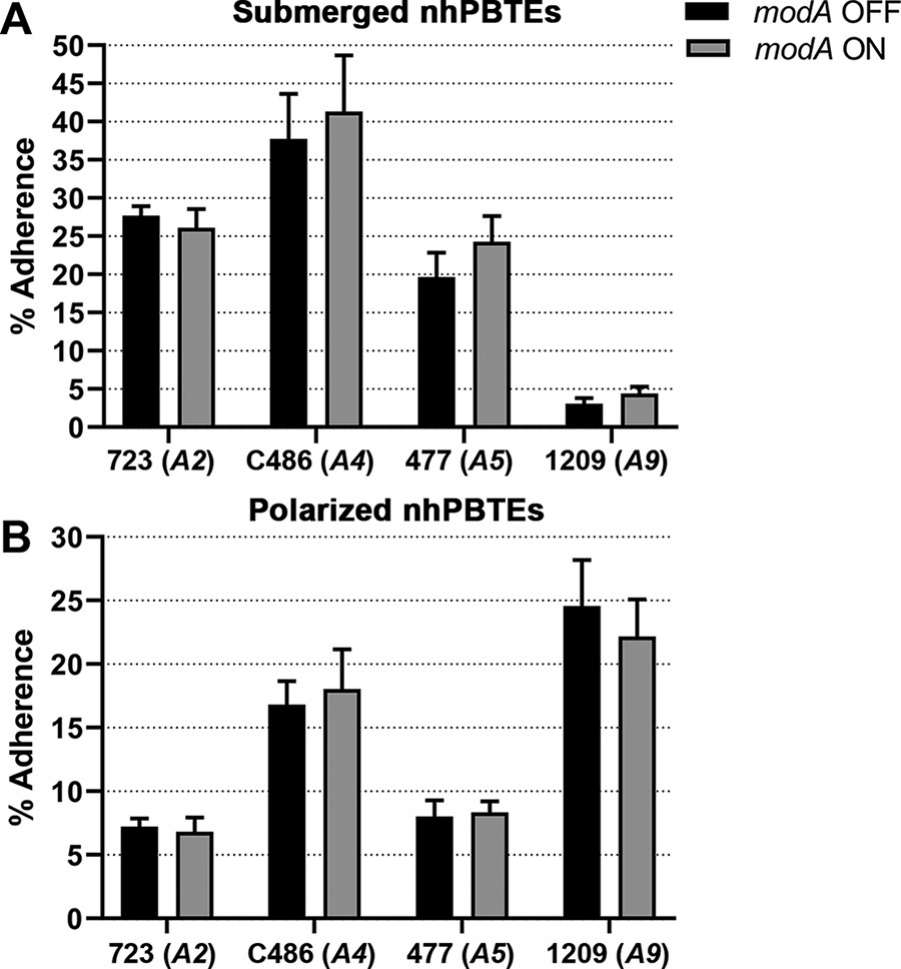
Adherence of NTHi *modA* locked variants to normal human primary bronchial-tracheal epithelial cells (nhPBTEs). Percentage of adherence to submerged nhPBTEs (A) or polarized nhPBTEs (B) after 1 h varied among strains; however, there was no significant difference between the *modA* variant pairs of any of the strains. (A) *modA2*, *n* = 24; *modA4*, *n* = 12; *modA5*, *n* = 9; *modA9*, *n* = 9. (B) *modA2*, *n* = 12; *modA4*, *n* = 9; *modA5*, *n* = 13; *modA9*, *n* = 15.

Following study of the role of ModA in the adherence to mucus, we assessed the ability of NTHi to adhere to nhPBTEs grown as monolayers submerged in culture medium. When grown submerged in medium, these cells do not differentiate into ciliated or mucus-producing goblet cells characteristic of the airway epithelium (41). However, these cells may represent the epithelial cells of the human respiratory tract and have been previously used to study NTHi adherence (42). Each strain adhered to submerged nhPBTEs to a different extent; of these strains, strain 1209 adhered the least (Fig. 2A), possibly due to general reduced expression of adhesins critical to adherence to these cells (33). No differences in adherence to submerged nhBPTEs was observed with any of the *modA* variant pairs.

Submerged nhPBTEs are not representative of the complex pseudostratified epithelium found in the airways. Therefore, nhPBTEs were grown at the air-liquid interface (ALI) and allowed to differentiate into ciliated cells and mucus-producing goblet cells. To assess the adherence of NTHi strains to pseudostratified respiratory tract epithelium, mucus was rinsed from the apical surface of the polarized nhPBTEs, and individual *modA* variants were allowed to adhere to the polarized cells for 1 h. There was no significant difference between the adherence of any *modA* variant pairs. However, a ModA-independent difference was observed between the strains tested, as strains C486 and 1209 appeared to adhere better to the surface of polarized nhPBTEs in comparison to strains 723 and 477 (Fig. 2B).

This contrasted the observation that strain 1209 was the least adherent to submerged nhPBTEs, suggesting that different adhesins may be required for adherence to submerged and polarized nhPBTEs potentially because of the difference in composition of cells and presence of residual mucus on the polarized nhPBTEs. There was no statistically significant difference observed between any of the *modA* variant pairs for any of the strains tested (Fig. 2A and B). Therefore, ModA2, ModA4, ModA5, and ModA9 likely do not regulate factors important for the adherence of NTHi to submerged or differentiated nhPBTEs, which are much more representative of the respiratory tract epithelium present *in vivo*.

### ModA2 and ModA9 regulate adherence to middle ear epithelial cells.

**FIG 3:**
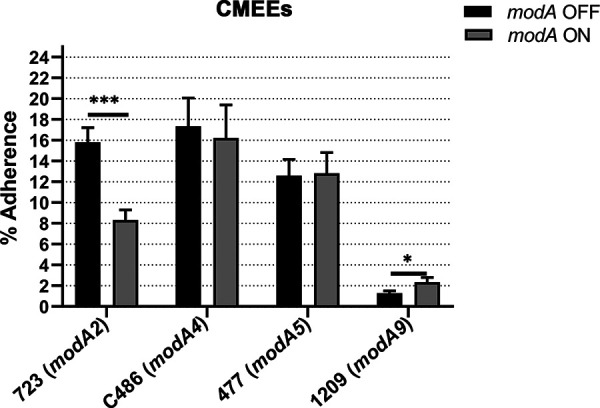
Adherence of NTHi *modA2* locked variants to chinchilla middle ear epithelial cells (CMEEs). The data are shown as percentages of adherent bacteria relative to inoculum after 1 h. *modA2* OFF adhered significantly better than *modA2* ON and *modA9* ON adhered significantly better than *modA9* OFF. *modA2*, *n* = 10; *modA4*, *n* = 13; *modA5*, *n* = 13; *modA9*, *n* = 21. *, *P < *0.05; ***, *P < *0.001 (Student’s *t* test).

As the middle ear is a major site for NTHi infection, the middle ear epithelium is a common substrate for adherence by NTHi. The chinchilla is an established animal model to study the course of acute OM (43), and human middle ear cell lines are not readily available; therefore, chinchilla middle ear epithelial cells (CMEEs) were selected to study the adherence of NTHi to middle ear epithelium. CMEEs were cultured submerged in medium until confluent monolayers were formed. These monolayers lack ciliated cells and more closely resemble the squamous epithelial cells found within the middle ear epithelium (44). Adherence to CMEEs was similar for both variants of strains C486 (ModA4) and 477 (ModA5), with no ModA-dependent differences observed (Fig. 3). However, a ModA-dependent difference was observed with strains 723 (ModA2) and 1209 (ModA9). In strain 723, the *modA2* OFF variant adhered to CMEEs significantly better than the *modA2* ON variant (Fig. 3; *P* < 0.001), in direct contrast to the phenotype observed with mucus (Fig. 1). For strain 1209, the *modA9* OFF variant adhered significantly less than the *modA9* ON variant (Fig. 3; *P* < 0.05), although strain 1209 was overall less adherent to CMEEs than the other three strains assessed. Thus, it appears that ModA2 and ModA9 regulate factors required for the adherence of NTHi to middle ear epithelial cells in distinct ways, although the exact nature of these factors is yet to be determined.

### ModA2 and ModA9 regulate factors required for adherence to vitronectin.

**FIG 4:**
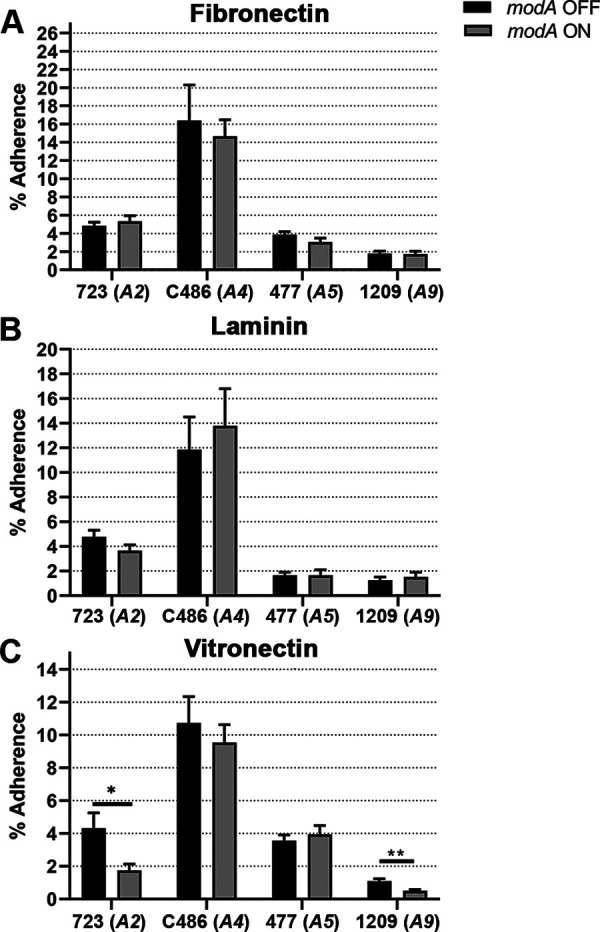
Adherence of NTHi *modA* locked variants to extracellular matrix components. The percentage of adherence to fibronectin (A), laminin (B), and vitronectin (C) after 1 h was plotted. Strain-specific differences in adherence were observed for all ECM components. There were no ModA-specific differences in adherence to fibronectin or laminin for any of the strains. Adherence to vitronectin was significantly different between the *modA2* and *modA9* variant pairs. (A) *modA2*, *n* = 10; *modA4*, *n* = 11; *modA5*, *n* = 12; *modA9*, *n* = 16. (B) *modA2*, *n* = 10; *modA4*, *n* = 12; *modA5*, *n* = 12; *modA9*, *n* = 16. (C) *modA2*, *n* = 9; *modA4*, *n* = 12; *modA5*, *n* = 16; *modA9*, *n* = 12. *, *P < *0.05; **, *P < *0.01 (Student’s *t* test).

Adherence of NTHi to extracellular matrix (ECM) components is important for adherence to host mucosal surfaces and survival of the pathogen during disease (42, 45–47). Therefore, adherence of NTHi to the ECM components fibronectin, laminin, and vitronectin was assessed. *modA* status did not affect the adherence of any of the variant pairs to fibronectin or laminin (Fig. 4A and B); however, a significant difference in adherence to vitronectin was observed with strains 723 (*modA2*) and 1209 (*modA9*). Both the *modA2* OFF and *modA9* OFF variants adhered significantly better to vitronectin than their *modA* ON counterparts (Fig. 4C; *P* = 0.02 and *P* = 0.001, respectively). For strain 1209, this was the reverse of the phenotype observed for CMEEs (*modA9* ON adhered better to CMEEs than *modA9* OFF; compare with Fig. 3). Notably, strain 1209 adhered the least to all three ECM components, whereas strain C486 adhered the most (Fig. 4A to C). Adherence of the *modA2* variants to vitronectin was also assessed by microscopy (Fig. S1), which showed that *modA2* OFF bound more than *modA2* ON to vitronectin immobilized on a glass slide, whereas both variants bound equivalently and significantly less to bovine serum albumin (BSA) (Fig. S1). This agreed with the higher percentage of adherence of *modA2* OFF to vitronectin than *modA2* ON by CFU (Fig. 4C). Overall, these results suggest that ModA2 and ModA9 phasevarions regulate factors required for the adherence of NTHi to vitronectin.

### Regulation of adherence by ModA2 is partially dependent on the PE and P4 adhesins.

Of the various substrates tested, a clear correlation was observed in adherence of the *modA2* variants of NTHi strain 723 to vitronectin and CMEEs, where the *modA2* OFF variant adhered significantly better than the *modA2* ON variant. Moreover, the reduction in adherence of the *modA2* ON variant to these substrates was not due to a growth defect as shown by equivalent growth rates of both variants in brain-heart infusion broth supplemented with 2 μg/mL hemin and 2 μg/mL β-NAD (sBHI) and 1× Dulbecco’s phosphate-buffered saline (DPBS; medium used for assay of adherence to vitronectin) and the higher growth rate of the *modA2* ON variant in CMEE GM (medium used for assay of adherence to CMEEs) (Fig. S2). Therefore, several NTHi adhesins previously characterized as being required for adherence to vitronectin and CMEEs were investigated.

**FIG 5:**
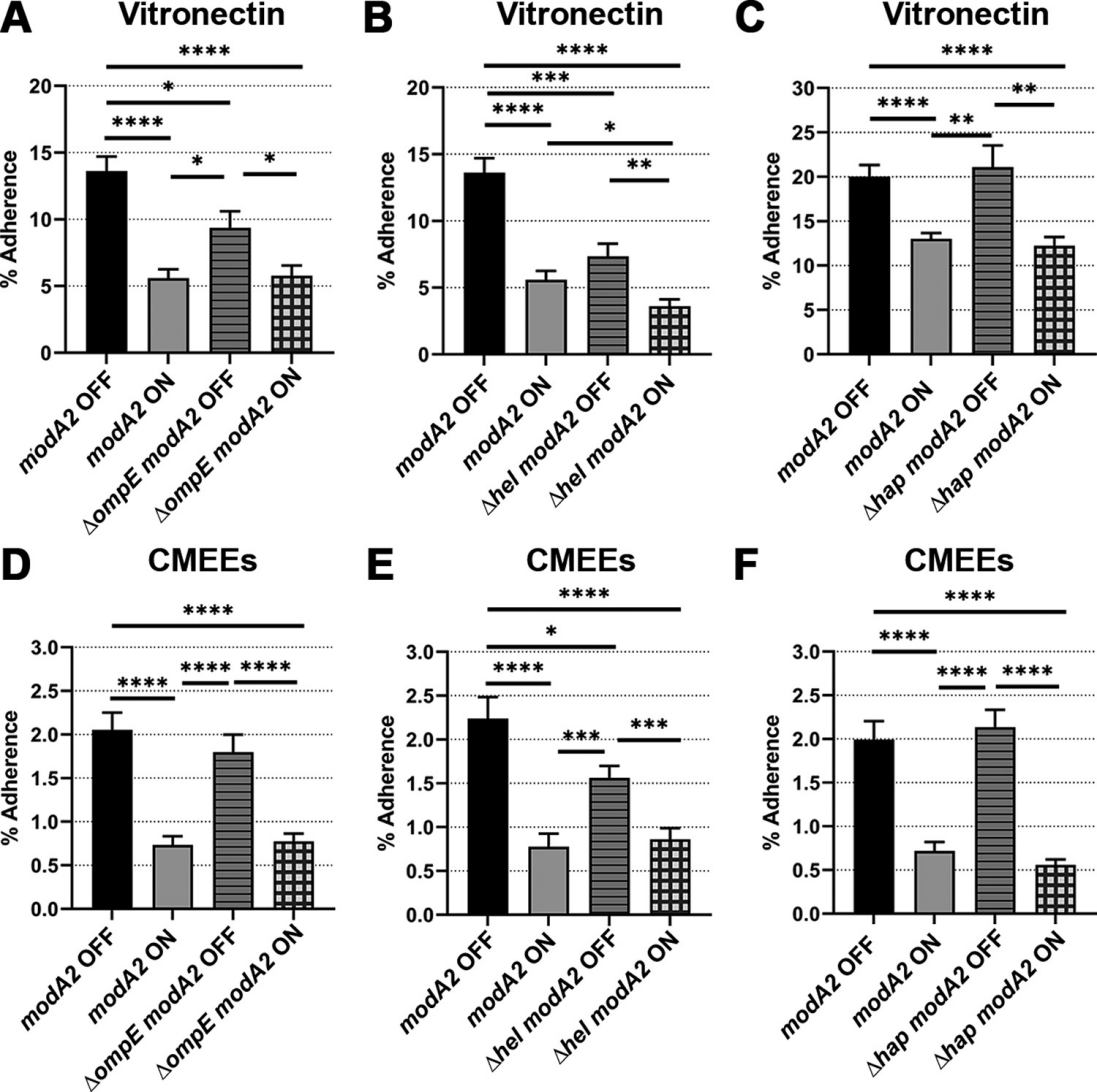
Role of adhesins PE (*ompE*), P4 (*hel*), and Hap (*hap*) in ModA2-dependent adherence of NTHi to vitronectin and CMEEs. Wild-type and adhesin mutants of *modA2* OFF and *modA2* ON of strain 723 were compared for adherence to vitronectin and CMEEs. (A) Deletion of *ompE* significantly reduced the adherence of *modA2* OFF to vitronectin but did not affect *modA2* ON. (B) Deletion of *hel* reduced the adherence of *modA2* OFF to vitronectin more significantly than that of *modA2* ON. (D, E) Deletion of *ompE* did not affect the adherence of either variant to CMEEs (D), whereas deletion of *hel* significantly reduced adherence of *modA2* OFF and not *modA2* ON (E). (C, F) Deletion of *hap* did not affect the adherence of the *modA2* variants to vitronectin (C) and CMEEs (F). (A) *n* = 11. (B) *n* = 11. (C) *n* = 11. (D) *n* = 17. (E) *n* = 17. (F) *n* = 11. *, *P* < 0.05; **, *P* < 0.01; ***, *P* < 0.001; and ****, *P* < 0.0001 (Student’s *t* test).

The adhesin Protein E (PE) mediates adherence of NTHi to vitronectin in addition to epithelial cells (42, 47). The gene that codes for PE, *ompE*, was deleted from the genome of the *modA2* locked variants. Deletion of *ompE* significantly reduced adherence of the *modA2* OFF variant to vitronectin (Fig. 5A; *P* < 0.05) but did not alter adherence of the *modA2* ON variant (Fig. 5A; *P* = 0.85). Since loss of *ompE* affected only the *modA2* OFF variant, PE likely contributes to ModA2-dependent regulation of adherence to vitronectin. However, deletion of *ompE* from the *modA2* OFF variant did not completely reduce adherence to that of the *modA2* ON variant. Therefore, PE may not be the only factor involved in the increased adherence of *modA2* OFF to vitronectin. The surface-associated lipoprotein and adhesin, P4 (or outer membrane protein 4), encoded by the gene *hel*, has also been shown to mediate adherence of NTHi to vitronectin and is required for survival of NTHi in the middle ear (47). Deletion of *hel* significantly reduced the adherence of both variants to vitronectin (Fig. 5B). The adherence of the *modA2* OFF variant was reduced by 47% (Fig. 5B; 13.6% to 7.3%; *P* < 0.001), whereas that of the *modA2* ON variant reduced by only 35% (Fig. 5B; 5.5 to 3.6%; *P* < 0.05). This result suggested that loss of P4 affected the *modA2* OFF variant to a greater extent than the *modA2* ON variant, and therefore, P4 may contribute to both ModA2-dependent and ModA2-independent regulation of adherence to vitronectin. The role of Hap, an autotransporter protein and known adhesin (45), was also investigated in ModA2-regulated adherence to vitronectin. Deletion of *hap* did not affect adherence to vitronectin (Fig. 5C), which was expected since Hap is known to bind to the ECM components fibronectin, laminin, and collagen IV but not vitronectin (45, 47).

Next, adherence of the adhesin mutants to CMEEs was assessed and compared with that of the parental variants. There was no effect of *ompE* deletion on the adherence of either variant to CMEEs (Fig. 5D), and this suggested that PE may not be essential for adherence of strain 723 to CMEEs in these assays. In contrast, deletion of *hel* significantly reduced adherence of the *modA2* OFF variant to CMEEs but did not affect adherence of the *modA2* ON variant (Fig. 5E), which suggested that P4 may contribute to ModA2-dependent regulation of adherence to CMEEs. There was no significant difference between adherence of the *hap* mutant variants and the wild-type variants to CMEEs (Fig. 5F). Therefore, Hap may not contribute to the ModA2-dependent regulation of adherence to CMEEs. Taken together, these data suggest that both PE and P4 are involved in the ModA2-dependent regulation of adherence to vitronectin and that P4 contributes to the ModA2-dependent regulation of adherence to CMEEs.

## DISCUSSION

Adherence of NTHi to the host airway is critical for colonization as a commensal as well as for pathogenesis. Since different ModA phasevarions regulate the expression of multiple different surface-associated proteins, including adhesins (33), and affect various aspects of pathogenesis of NTHi (33–36), this study aimed to address the role of distinct ModA phasevarions in the adherence of NTHi to host airway components. The *modA* alleles *modA2*, *modA4*, *modA5*, *modA9*, and *modA10* are the most prevalent alleles in clinical isolates of NTHi collected from the nasopharynx of healthy individuals and the middle ears of OM patients, whereas the *modA2*, *modA4*, and *modA5* alleles are highly prevalent in the lungs of COPD patients (32, 33). Since the role of ModA10 in NTHi adherence to respiratory epithelial cells has already been studied (48), the ModA2, ModA4, ModA5, and ModA9 phasevarions were selected for this study and are represented by the strains 723, C486, 477, and 1209, respectively. The use of a single strain for each *modA* allele may be a limitation of the study, but the strains selected have been well characterized in the literature and are the only strains for which locked, or non-phase-variable, variant pairs are available. Variants of each of the four strains with the *modA* status locked to either OFF or ON allowed for assessment of the direct effect of each specific *modA* status. The adherence of the variant pairs of all four strains to different cellular and noncellular substrates that are commonly encountered by NTHi within the airway was assessed.

This study demonstrated that the status of *modA2*, the most prevalent *modA* allele (33), significantly affected the adherence of NTHi to diverse respiratory tract substrates. The *modA2* ON variant of strain 723 adhered better to mucus, whereas the *modA2* OFF variant adhered better to CMEEs and vitronectin. Mucus is present throughout the airways and serves as a substrate for NTHi adherence during both colonization and disease. CMEEs isolated from the healthy chinchilla ear were used to represent epithelial cells encountered by the pathogen during middle ear infection. The lack of a human middle ear epithelium presents another limitation of this study and may limit the translation of these findings to human middle ear epithelium. However, as cell lines derived from healthy and diseased human subjects become more readily available, the results presented herein could be extrapolated to future studies that utilize human middle ear epithelial cells. The ECM component vitronectin is detected in numerous parts of the airway, including the middle ear basement membrane, bronchial epithelium, and bronchial submucosal glands (49, 50), which may become available to NTHi during disease. This implies that a dynamic set of factors are regulated by the ModA2 phasevarion, each contributing to adherence to distinct ligands found in the human airway. The ModA9 phasevarion also differentially regulated factors required for adherence to CMEEs and vitronectin but with a different phenotypic pattern compared to strain 723. As *modA9* is most prevalent in OM isolates and not in COPD isolates (32, 33), the regulation of adherence by ModA9 may be of importance in the pathogenesis of NTHi during OM. In addition, strain 1209 (ModA9) adhered better to mucus and polarized epithelial cells than to submerged epithelial cells and ECM components, irrespective of *modA9* status, which suggests that additional, non-ModA-regulated factors contribute to the phenotypes observed in this strain and add an additional level of complexity to the regulation of adherence. Therefore, different ModA phasevarions affected the adherence of NTHi to different respiratory substrates in a unique and complex manner, suggesting cell- and niche-specific advantages conferred by the particular genes regulated by each phasevarion, in addition to differences exhibited by different strains irrespective of their *modA* status.

NTHi frequently encounter and adhere to mucus on the epithelial surface within the nasopharynx during colonization, as well as at the sites of disease. The ascension of NTHi from the nasopharynx to the middle ear occurs via adherence to mucus within the Eustachian tube lumen. The movement of mucus and bacteria into the middle ear commonly occurs when the upper airway is compromised by viral infection, which leads to changes in the abundance and composition of mucus (19). Thus, ModA phasevarions that regulate adherence to mucus may play a role in adaptation of NTHi within the nasopharynx during conditions such as viral infections and contribute to subsequent survival and selection within other sites in the airway. Since the ModA2, ModA4, and ModA9 phasevarions are reported to be prevalent in nasopharyngeal isolates recovered from healthy individuals (33), they may play a role in asymptomatic colonization of the nasopharynx. Although adherence of NTHi to ciliated epithelium was not regulated by these phasevarions, they may nonetheless affect colonization by alternate mechanisms. As the *modA2* ON variant adhered to mucus better than the *modA2* OFF variant, the *modA2* ON status may be more advantageous for NTHi during initial colonization, as well as during ascension from the nasopharynx to the middle ear. We previously reported that the *modA2* ON status is selected for within middle ear fluids in a chinchilla model of experimental otitis media and that a shift from *modA2* OFF to ON status occurs within the middle ear that leads to a more severe disease pathology than when the middle ear is initially challenged with a predominantly *modA2* ON population (33, 36). Overall, the data presented herein suggest that NTHi may enter the middle ear predominantly in the *modA2* ON status following ascension from the nasopharynx, due in part to increased adherence to mucus.

Adherence to vitronectin depended differently on the adhesins PE and P4. While PE may contribute to ModA2-dependent regulation of adherence to vitronectin, P4 may participate in ModA2-dependent regulation of adherence to middle ear epithelial cells, as well as vitronectin. This agrees with the role of P4 in the virulence of NTHi in the middle ear (47). PE and P4 are also known to bind to fibronectin and laminin (47). However, ModA2 did not affect adherence to fibronectin or laminin. Similarly, *modA2* status did not affect adherence to bronchial epithelial cells, a process known to be dependent on PE (42). This could be due to the involvement of other bacterial and host factors (21, 33, 51) and yet-to-be-identified effects of the ModA2 phasevarion. Interestingly, the transcription of *ompE* and *hel* has been found to be unaffected by the status of *modA2* (33), suggesting a role for ModA2 in the presentation or accessibility of these adhesins on the surface of NTHi.

This study established that clinically prevalent ModA phasevarions regulate adherence of NTHi to specific host airway substrates. These findings are important because many of these adhesins have been investigated as candidates for a subunit NTHi vaccine (52–56). Adhesins have also been useful in the diagnosis of NTHi-induced respiratory infections (57) and can be targeted for eradication of adherent NTHi from the site of disease (58). Characterization of strains expressing distinct ModA allelic variants has demonstrated that under certain environmental conditions, either *modA* OFF or *modA* ON variants give the strain distinct advantages. Therefore, the switch from *modA* OFF to *modA* ON, or vice versa, may enable NTHi to evade recognition and clearance by the host immune response or therapeutic agents. As such, an understanding of the mode of regulation of adherence by each ModA phasevarion at the site of colonization and disease is necessary to validate the use of the regulated adhesins as vaccine candidates, diagnostic tools, and therapeutic agents against NTHi.

## MATERIALS AND METHODS

### Bacterial strains and growth conditions.

NTHi strains 723, 477, and 1209 were received from the Finnish Otitis Media study group (59), and strain C486 was isolated from a child with otitis media (60). The *modA* locked variants of each of these strains were constructed as described previously (34), so that these strains were unable to switch the status of *modA.* NTHi strains were cultured at 37°C and 5% CO_2_ on chocolate agar or in sBHI. All of the strains used are listed in Table 1.

### Generation of mutants.

DNA fragments containing a kanamycin resistance gene flanked by sequences homologous to the sequences flanking the target genes were designed and then synthesized (Integrated DNA Technologies). Each fragment was ligated into a pJET1.2 blunt end cloning vector at the EcoRV restriction site using a CloneJET PCR cloning kit (Thermo Scientific). Escherichia coli DH10B competent cells were transformed with the ligation products, and transformant colonies were selected on LB agar plates containing ampicillin. Plasmids isolated from these transformant colonies were used as the templates for amplifying the inserts using the pJET1.2 forward and reverse sequencing primers (Table 2). NTHi 723 *modA2* locked OFF and *modA2* locked ON variant strains were transformed with the amplified inserts using the M-IV method (61). NTHi transformants were selected on chocolate agar plates supplemented with kanamycin. The mutants were confirmed by sequencing, as well as by PCR using kanamycin resistance cassette internal primers and primers for flanking sequences of the target gene. All of the primers used for cloning and PCR are listed in Table 2.

**TABLE 2:** Primers

Primer name	Sequence
pJET1.2 forward	5′-CGACTCACTATAGGGAGAGCGGC-3′
pJET1.2 reverse	5′-AAGAACATCGATTTTCCATGGCAG-3′
*ompE* confirmatory forward	5′-CCTAGAAGGTTATGGGCACACTG-3′
*ompE* confirmatory reverse	5′-GCCAGCAGTAAAATAGCAATAACTGC-3′
*hel* confirmatory forward	5′-CGACCTGCCGCATAAACATTTGG-3′
*hel* confirmatory reverse	5′-GACGAAGACCCAATTCACGAGC-3′
*hap* confirmatory forward	5′-ACCGCAGACTGGATTGTCGATC-3′
*hap* confirmatory reverse	5′-GCAATAATGCCATCGCCCACAC-3′
Kan^r^ cassette internal forward	5′-GCACCTGATTGCCCGACATTATC-3′
Kan^r^ cassette internal reverse	5′-GGACGAGTCGGAATCGCAGAC-3′

### Isolation and culturing of CMEEs.

Chinchilla middle ear tissues (mucosa of the inferior bullae) were aseptically harvested from naive animals and cultured in explant medium (62) containing DMEM (Mediatech), Ham’s F-12 (Mediatech), glutamine (Mediatech), hydrocortisone (Stemcell Technologies), isoproterenol (Sigma), and fetal bovine serum (FBS) (Mediatech). After generation of epithelial cells (CMEEs) from the tissues, the tissue explants were removed. CMEEs were subcultured and maintained submerged in explant medium containing epidermal growth factor (EGF) (Sigma), i.e., CMEE growth medium.

### Adherence assays with CMEEs.

CMEEs were seeded into wells of 96-well, flat-bottomed plates (Costar) and maintained submerged in culture medium until the formation of a tightly packed monolayer. Bacterial inoculum was prepared from log phase cultures of NTHi variant pairs and added to wells containing the CMEEs at an multiplicity of infection (MOI) of 100. The cells were incubated at 37°C and 5% CO_2_ for 1 h to allow the bacteria to adhere. The medium was removed and the CMEEs were washed three times with 1× DPBS to remove nonadherent bacteria. Then, 10× TrypLE (Gibco) was used to detach the adherent bacteria, which were then collected in 1× DPBS, serially diluted, and plated on chocolate agar. The percentage of adherence was determined from the CFU values of the adherent bacteria and the inoculum. The experiment was carried out at least three times with a minimum of three biological replicates each time.

### Adherence assays with submerged nhPBTEs.

Normal human primary bronchial-tracheal epithelial cells (nhPBTEs) obtained from healthy human lungs (ATCC PCS-300-010) were seeded into the wells of 96-well, flat-bottomed plates (Costar) and maintained in Pneuma-Cult expansion medium (PC-Ex) (Stemcell Technologies) until confluence. Bacterial inoculum was prepared from log phase cultures of NTHi and added to wells containing the nhPBTEs at an MOI of 100. The cells were incubated for 1 h at 37°C and 5% CO_2_. The medium was removed, and the cells were washed three times with 1× DPBS to remove nonadherent bacteria, and 10× TrypLE (Gibco) was used to detach the adherent bacteria, which were then collected in 1× DPBS and plated on chocolate agar. The percentage of adherence was determined as described above. The experiment was carried out at least three times with a minimum of three biological replicates each time.

### Adherence assays with polarized nhPBTEs.

nhPBTEs were seeded in 6.5-mm Transwells (Corning Transwells) and maintained in PC-Ex expansion medium. Upon reaching confluence, the medium was removed from the apical surface, and the cells were fed basolaterally with Pneuma-Cult ALI (air-liquid interface) differentiation medium (Stemcell Technologies) for 5 to 8 weeks to allow differentiation of cells at the air-liquid interface. For the assay, the apical surface was washed with 1× DPBS to remove the existing mucus produced by these cells. Bacterial inoculum was prepared from a log phase culture of NTHi, added to the cells at an MOI of 100 and incubated at 37°C and 5% CO_2_ for 1 h. After the incubation period, the supernatant was removed, and the apical surface was washed three times with 1× DPBS to remove nonadherent bacteria. 10× TrypLE (Gibco) was added to the apical surface, and 1× TrypLE was added to the basolateral surface to dissociate the adherent bacteria. Samples were collected in 1× DPBS and plated on chocolate agar. The percentage of adherence was determined as described above. The experiment was carried out at least three times with a minimum of three biological replicates each time.

### Adherence assays with mucus.

nhPBTEs were differentiated into polarized cells at the air-liquid interface as described above and cultured until mucus production was observed. The apical surface was incubated with 1× DPBS at 37°C for 15 min, and the mucus was collected by pipetting. The mucus was quantified using a Qubit protein assay kit and then added into the wells of Nunc MaxiSorp flat-bottomed, 96-well plates at a concentration of 10 μg/well and incubated overnight at 37°C. Prior to the assay, mucus-coated wells were washed four times with 1× DPBS to remove excess mucus. Bacterial inoculum was prepared from log phase cultures of NTHi in 1× DPBS and added at a density of 5 × 10^6^ CFU/well. After 1 h of incubation at 37°C and 5% CO_2_, the supernatant was removed, and the wells were washed four times with 1× DPBS to remove nonadherent bacteria. Then, 1× DPBS (100 μL) was added to each well, and the adherent bacteria were dislodged and collected by vigorous pipetting and scraping of the wells. Dilutions of the collected samples were plated on chocolate agar. The percentage of adherence was determined using CFU values as described above. The experiment was carried out at least three times with a minimum of three biological replicates each time.

### Adherence assays with ECM components.

Flat-bottomed, 96-well tissue culture-treated plates (Costar) were coated with fibronectin (Sigma-Aldrich), laminin (Sigma-Aldrich), or vitronectin (Sigma-Aldrich) according to manufacturer protocols. Briefly, working solutions of vitronectin (1.5 μg/mL) and laminin (6 μg/mL) were prepared in 1× DPBS, whereas fibronectin was reconstituted in water (15 μg/mL). A total of 100 μL of the working stock of vitronectin was added per well of 96-well plate and incubated at 37°C for 2 h followed by overnight incubation at 4°C, and 100 μL of the working stocks of laminin and fibronectin were added per well on the day of the assay, removed immediately, and allowed to dry. The coated wells were washed twice with 1× DPBS just prior to the assay. Bacterial inoculum was prepared from log phase cultures of NTHi and added to coated wells at a density of 5 × 10^6^ CFU/well. After incubation at 37°C and 5% CO_2_ for 1 h, the supernatant was removed, and the wells were washed four times with 1× DPBS to remove any nonadherent bacteria. Adherent bacteria were collected in 100 μL 1× DPBS with vigorous pipetting and scraping of the wells. The collected samples were serially diluted and plated on chocolate agar. The percentage of adherence was determined as described above. The experiment was carried out at least three times with a minimum of three biological replicates each time.

### Assessment of adherence to vitronectin by microscopy.

A previously established method (63) was modified to assess adherence of NTHi to a vitronectin-coated glass surface by microscopy. A solution of vitronectin (Recombinant human VTN, Gibco A14700) was prepared in 1× DPBS at the concentration of 2 μg/mL. Then, 10 μL was added as a drop onto a glass slide and allowed to dry for 30 min at room temperature. Similarly, a glass slide was coated with 1% BSA as a negative control. The slides were washed three times by dipping for 2 s in 1× DPBS to remove excess protein. Bacterial inoculum was prepared from log phase cultures of NTHi in 1× DPBS at a density of 1 × 10^8^ CFU/mL. The coated glass slides were submerged in 10 mL of bacterial inoculum in sterile petri dishes and incubated at 37°C, and 5% CO_2_ for 1 h at 25 rpm. After incubation, the slides were washed three times in 1× DPBS to remove any unbound bacteria. The slides were air-dried, heat-fixed by passing over a flame, and stained with methylene blue (Epredia Shandon Kwik-Diff Stains) for 60 s. Excess stain was removed by washing gently with water. The slides were air-dried and imaged using the 20× objective of a Zeiss Axioscope 5 microscope. The images were processed using ZEN 3.0 software, and the area occupied by NTHi in each field of view was determined with ImageJ software. The experiment was carried out three times with at least five fields imaged for each sample.

### Growth assessment.

Log phase cultures of NTHi 723 *modA2* variants were added to 200 μL of sBHI, CMEE GM or 1× DPBS at 1 × 10^7^ CFU, 1 × 10^7^ CFU, and 2 × 10^8^ CFU, respectively, in a 96-well, flat-bottomed plate (Costar). The cultures were incubated at 37°C and 5% CO_2_, and optical density at 490 nm (OD_490_) was measured at the interval of 15 min for the duration of 1 h in a Biotek Synergy microplate reader. Media without bacterial culture were used as blank. OD_490_ of blank were subtracted from that of the samples for each time point and plotted. The experiment was carried out at least three times with a minimum of three biological replicates each time.

### Statistical analysis.

Statistical significance was assessed by Student’s unpaired *t* test using GraphPad Prism software, version 8.4.2. A *P* value less than or equal to 0.05 was considered statistically significant.
