# Supplementary material for: Adherence of Nontypeable Haemophilus influenzae to Cells and Substrates of the Airway Is Differentially Regulated by Individual ModA Phasevarions
Source: Microbiol Spectr. 2022 Dec 13;11(1):e04093-22. doi: 10.1128/spectrum.04093-22 (PMC9927368; doi:10.1128/spectrum.04093-22)
Supplement: Supplemental file 1 — Supplemental material. Download spectrum.04093-22-s0001.pdf, PDF file, 0.3 MB [file spectrum.04093-22-s0001.pdf]

Supplemental Information

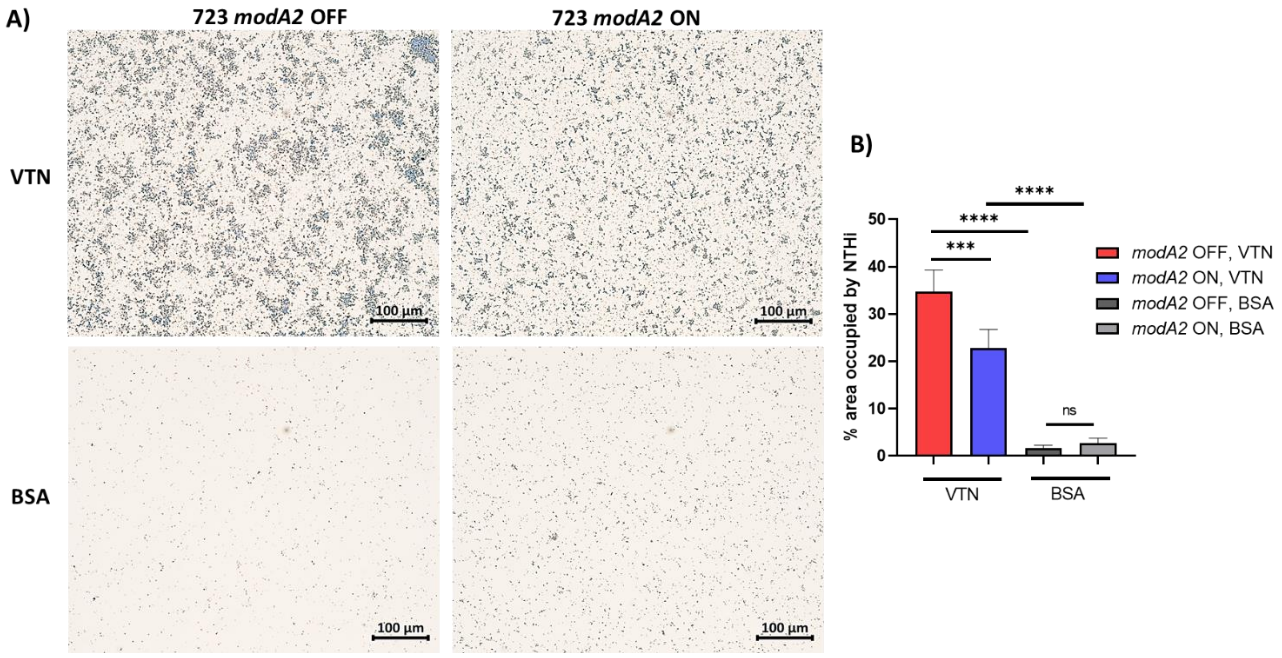

Fig. S1. Adherence of NTHi 723 *modA2* locked variants to vitronectin. A) NTHi strain 723 *modA2* locked variants were allowed to adhere to glass slides coated with vitronectin (VTN) or BSA, as a control. Adherent NTHi were visualized by staining with methylene blue and imaging with bright field microscopy. Representative fields with the respective scale bar are shown. **B)** The percent area occupied by adherent NTHi per field was plotted ($n \geq 5$). The *modA2* OFF variant adhered significantly more to vitronectin than the *modA2* ON variant, whereas both variants adhered equivalently and less to BSA. Representative data from a single experiment (out of 3) are presented. *** $p < 0.001$, **** p-value < 0.0001 and 'ns' is non-significant, Student's t test.

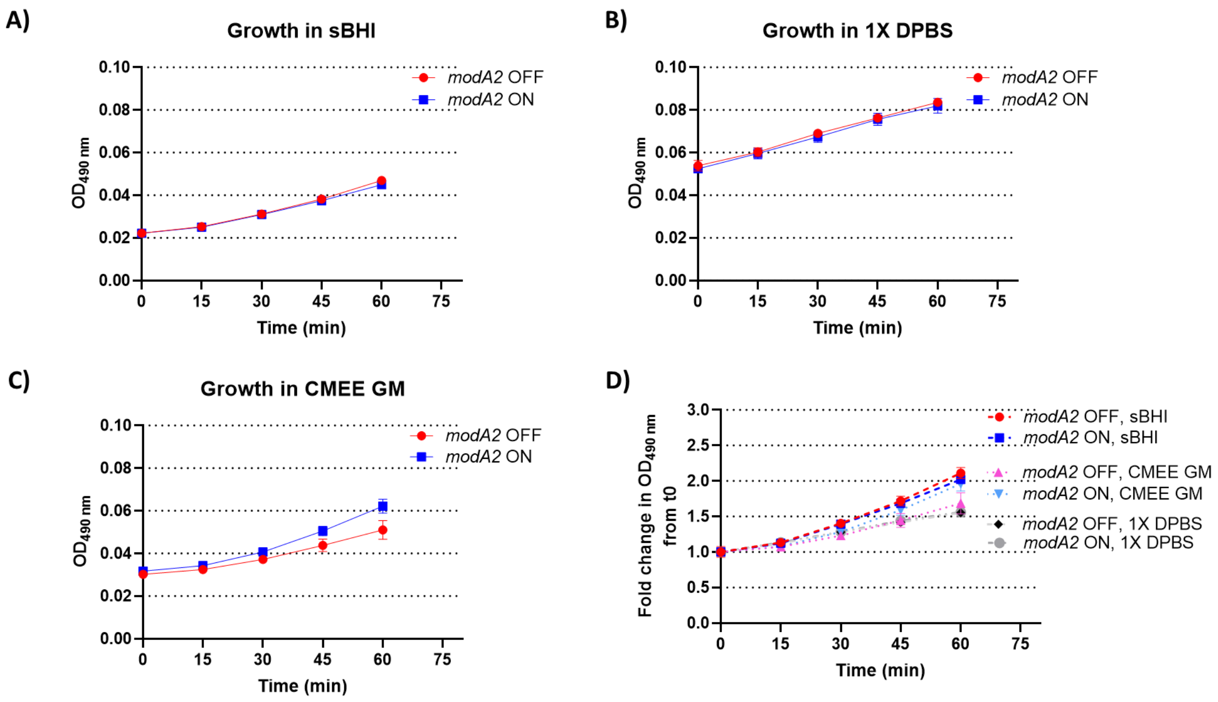

Fig. S2. Growth of the *modA2* variants. Growth of *modA2* OFF and *modA2* ON was assessed by measuring the optical density (OD) at 490 nm of bacterial cultures growing in **A)** sBHI **B)** 1X DPBS and **C)** CMEE growth medium (CMEE GM) for 1 hour. Data are shown as OD_{490nm} of samples normalized to the respective blank medium. **D)** Fold change in growth from inoculation is plotted wherein both variants showed equivalent growth in sBHI and 1X DPBS, and *modA2* ON (light blue) had an increased growth rate in CMEE GM compared to *modA2* OFF (pink). Data from a single experiment (out of 3) with 3 biological replicates (n=3) are represented here.
